# Persistent adverse effects of antidepressants

**DOI:** 10.1017/S2045796019000520

**Published:** 2019-09-23

**Authors:** Joanna Moncrieff

**Affiliations:** Division of Psychiatry, University College London, Gower street, London, WC1E 6BT, UK

**Keywords:** Adverse effects, antidepressants, drug side effects other, psychotropic drugs, sexual dysfunction

Antidepressants have been prescribed to millions of people worldwide on the unproven assumption that depression is caused or mediated by specific abnormalities of brain chemicals that antidepressants can correct, alongside evidence of marginal differences from placebo on depression rating scales in randomised trials. Yet, antidepressants are chemicals which alter the normal functioning of the brain and other parts of the body in ways we do not fully understand. It is becoming clear that far from normalising brain function, antidepressants disrupt normal biological processes with potentially devastating consequences for some people who take them.

We are not clear about the nature of the neurochemical and physiological changes that occur immediately after taking an antidepressant, nor how they vary across the different agents. We are even less certain about how the body, including the brain, adapts to the long-term presence of these drugs, and we do not know whether the alterations produced by the drugs return to normal once the drug is stopped, or whether they persist. Yet we know that other drugs used for mental health problems sometimes cause irreversible brain alterations, such as tardive dyskinesia caused by antipsychotics. The brain is a delicate organ; it may not take much to permanently re-set its structure or function.

Historically, the medical community has been slow to appreciate the extent to which drugs can interfere with normal brain and body functions. It took psychiatrists a long time to acknowledge that tardive dyskinesia was caused by antipsychotics (Moncrieff, 2013). It has taken three decades for the withdrawal effects of antidepressants to receive serious attention. The prescription opioid epidemic in the United States continues despite mounting evidence that the drugs can exacerbate chronic pain rather than relieve it (Velayudhan *et al*., 2014). Two editorials in this issue demonstrate how ‘legacy effects,’ that is effects that occur or persist after treatment ceases, seem to be a particular blind spot for both clinicians and researchers.

## Withdrawal effects

Withdrawal effects are, in themselves, an indication that the body has been altered by the ingestion of a drug. We associate withdrawal effects with long-term use, but in fact, the body can change, temporarily, even after a single dose of a drug. Animal studies show that one acute treatment with an opiate provokes a period of heightened sensitivity to pain (hyperalgesia), which follows after the direct analgesic effect of the drug and lasts for a few days (Celerier *et al*., 2000). Similarly, taking benzodiazepine hypnotics for just one or two days improves sleep initially, at least slightly, but when the pill is stopped, people find it even more difficult to sleep than they did before they took it (Soldatos *et al*., 1999). This situation is sometimes referred to as ‘rebound;’ a name for the compensatory-type effects that occur after the acute effects of a drug have worn off.

Following long-term use, some drugs give rise to more severe and longer-lasting withdrawal syndromes. Some are predictable ‘rebound’ type reactions (such as anxiety following benzodiazepine withdrawal), others, like tardive dyskinesia, are less predictable. Protracted withdrawal following benzodiazepine cessation was recognised back in 1991 with symptoms including anxiety, tinnitus and paraesthesia that lasted for many months and sometimes years. In most, though not all, instances there was a gradual improvement over time, but symptoms persisted in some cases, even despite the resumption of benzodiazepines (Ashton, 1991). Drugs like alcohol and opiates seem less likely to cause protracted withdrawal, but the hypersensitivity to pain that is a recognised feature of opiate withdrawal can take up to five months to normalise following detoxification (Treister *et al*., 2012).

Evidence on antidepressant withdrawal suggests a picture similar to the benzodiazepines. There is a range of intensity and duration, with not everyone experiencing debilitating or even noticeable symptoms, but there are numerous reports of withdrawal symptoms being severe and protracted. As Hengartner, Davies & Read conclude, withdrawal reactions associated with antidepressants are common, frequently severe and last from a few weeks to months and sometimes even longer (*Antidepressant withdrawal, the tide* is *finally turning*).

## Post-SSRI sexual dysfunction

Further evidence of lasting changes associated with antidepressants comes from the emerging literature on post-selective serotonin reuptake inhibitor (SSRI)-sexual dysfunction. SSRIs are well-known to impair sexual function while they are being taken, and Healy describes the accumulating evidence that difficulties can persist following cessation of the drugs for months and sometimes years (*post-SSRI sexual dysfunction and other enduring sexual dysfunctions*). Persistent sexual impairment is also demonstrated in male rats treated with SSRIs during adolescence (de Jong *et al*., 2006; Simonsen *et al*., 2016) and Healy documents how similar syndromes have been described following discontinuation of certain other drugs such as finasteride (prescribed for hair loss) and retinoids used for acne. It is difficult to estimate the prevalence of post-SSRI persistent sexual dysfunction given the currently limited data, but one survey identified that 34% of respondents showed evidence of possibly having the condition, and 4.3% showed a high probability of having it (Ben-Sheetrit *et al*., 2015). The fact that it is consistent with acute effects of SSRIs and with animal research supports the position that post-SSRI-sexual dysfunction is a genuine consequence of SSRI use rather than the re-emergence of symptoms of an underlying depression, as sometimes suggested. Like prolonged antidepressant withdrawal symptoms, Healy suggests that the usual course is for post-SSRI-sexual dysfunction to improve gradually, although possibly not in everyone.

## Implications

There is a large-scale failure to appreciate the risks involved in taking drugs that alter brain function on a long-term basis. Some of these risks are foreseeable, some less so. Given the history of benzodiazepines, we should have been able to anticipate that SSRIs and other new drugs for depression and anxiety would produce withdrawal syndromes, although once again we were taken unawares, and there seems to have been no research into this possibility before the drugs were launched. Syndromes like tardive dyskinesia, or opioid-induced hyperalgesia, should remind us, however, that the effects of drugs can be unexpected. The persistence of harmful effects also suggests that in some cases drugs can cause long-lasting changes.

The lack of systematic and well-funded research means that the prevalence of antidepressant withdrawal and post-SSRI sexual dysfunction remains uncertain, and the mechanisms obscure. It is estimated that around 13% of the population of England are taking antidepressants currently (Department of Health and Social Care (DHSC), 2018) and data from the United States from 2011–2014, put the figure at 12% (Pratt *et al*., 2017). If even a small proportion of these people experience protracted withdrawal or post-SSRI-sexual dysfunction, they amount to sizeable problems. The fact that it has taken single-minded and dedicated campaigners, many of them antidepressant users themselves, to bring these effects to the attention of the scientific and professional community must be received as a wake-up call.
